# Are all HCL systems the same? long term outcomes of three HCL systems in children with type 1 diabetes: real-life registry-based study

**DOI:** 10.3389/fendo.2023.1283181

**Published:** 2023-10-16

**Authors:** Alzbeta Santova, Lukas Plachy, Vit Neuman, Marketa Pavlikova, Lenka Petruzelkova, Petra Konecna, Petra Venhacova, Jaroslav Skvor, Renata Pomahacova, David Neumann, Jan Vosahlo, Jiri Strnadel, Kamila Kocourkova, Barbora Obermannova, Stepanka Pruhova, Ondrej Cinek, Zdenek Sumnik

**Affiliations:** ^1^ Department of Pediatrics, Motol University Hospital and 2^nd^ Faculty of Medicine, Prague, Czechia; ^2^ 1^st^ Faculty of Medicine, Charles University, Prague, Czechia; ^3^ Department of Probability and Mathematical Statistics, Faculty of Mathematics and Physics, Charles University, Prague, Czechia; ^4^ Department of Pediatrics, University Hospital Brno, Brno, Czechia; ^5^ Department of Pediatrics, University Hospital Olomouc, Olomouc, Czechia; ^6^ Department of Pediatrics, Masaryk Hospital, Usti nad Labem, Czechia; ^7^ Department of Pediatrics, University Hospital Plzen, Plzen, Czechia; ^8^ Department of Pediatrics, University Hospital Hradec Kralove, Hradec Kralove, Czechia; ^9^ Department of Pediatrics, University Hospital Kralovske Vinohrady, Prague, Czechia; ^10^ Department of Pediatrics, University Hospital Ostrava, Ostrava, Czechia; ^11^ Department of Pediatrics, Hospital Ceske Budejovice, Ceske Budejovice, Czechia

**Keywords:** type 1 diabetes, pediatrics, hybrid closed loop, AndroidAPS, registry

## Abstract

**Objective:**

To compare parameters of glycemic control among three types of hybrid closed loop (HCL) systems in children with T1D (CwD) using population-wide data from the national pediatric diabetes registry ČENDA.

**Methods:**

CwD aged <19 years treated with Medtronic MiniMed 780G (780G), Tandem t:slim X2 (Control-IQ) or do-it-yourself AndroidAPS (AAPS) systems for >12 months and monitored by CGM >70% of the time were included. HbA1c, times in glycemic ranges, and Glycemia Risk Index (GRI) were used for cross-sectional comparison between the HCL systems.

**Results:**

Data from 512 CwD were analyzed. 780G, Control-IQ and AAPS were used by 217 (42.4%), 211 (41.2%), and 84 (16.4%) CwD, respectively. The lowest HbA1c value was observed in the AAPS group (44 mmol/mol; IQR 8.0, p<0.0001 vs any other group), followed by Control-IQ and 780G groups (48 (IQR 11) and 52 (IQR 10) mmol/mol, respectively). All of the systems met the recommended criteria for time in range (78% in AAPS, 76% in 780G, and 75% in Control-IQ users). CwD using AAPS spent significantly more time in hypoglycemia (5% vs 2% in 780G and 3% in Control-IQ) and scored the highest GRI (32, IQR 17). The lowest GRI (27, IQR 15) was seen in 780G users.

**Conclusion:**

Although all HCL systems proved effective in maintaining recommended long-term glycemic control, we observed differences that illustrate strengths and weaknesses of particular systems. Our findings could help in individualizing the choice of HCL systems.

## 1 Introduction

Recent advances in technology increased the chance of optimizing long-term glycemic control in people with type 1 diabetes (T1D). The latest breakthrough is represented by the hybrid closed-loop (HCL) algorithms that can modify blood glucose level based on automated insulin dose adjustment (1). Growing evidence shows that HCL represent a safe and effective tool for the overall improvement of glycemic outcomes (2–6). Several randomized trials showed superiority of HCL over any other treatment modality in children and adults with T1D (7–11).

To date, there are several HCL systems available. Among them, Tandem t:slim X2 with Control-IQ algorithm (Tandem Diabetes Care, San Diego, CA, USA) and Medtronic MiniMed 780G with SmartGuard algorithm (Medtronic Inc. Minneapolis, MN, USA) are the ones most widely used in Europe. In addition, AndroidAPS (AAPS), an unofficial do-it-yourself (DIY) HCL system, continues to maintain significant popularity (12). Although all HCL systems share the same principle of manual pre-prandial bolus administration and automated insulin dose adjustment in case of predicted hypo- or hyperglycemia, there are also several differences mainly related to glycemic targets, reaction to hyperglycemia and user adjustable settings. Moreover, the systems differ in the used algorithm: whereas Control-IQ and AAPS use manually fully adjustable algorithms, 780G uses a self-adjusting technology that limits the users ability to influence insulin dosage. Although there are proofs of the efficacy to improve glycemic outcomes in each of these systems individually (3, 13, 14), studies directly comparing different HCL systems head-to-head in real-life settings are limited.

The aim of this study is to compare the parameters of glycemic control among the three most common types of HCL algorithms used in Czechia (MiniMed 780G with SmartGuard, Tandem t:slim X2 with Control-IQ and AAPS) in children with T1D (CwD) using the population-wide data from the national pediatric diabetes registry ČENDA.

## 2 Materials and methods

### 2.1 Study population and compared parameters

This retrospective multicenter study is based on data from the national pediatric diabetes web-based registry ČENDA, described in detail elsewhere (15). In brief, the registry stores anonymized data about CwD aged <19 years who are followed in one of the participating pediatric diabetes outpatient clinics in the Czech Republic. The data in this study are based exclusively on the annual report from 2022. Forty-seven pediatric diabetes outpatient clinics participated in ČENDA in 2022. As of December 2022, the ČENDA registry included 4427 CwD which is estimated to be more than 95% of all pediatric diabetes cases in the Czech Republic. Participation in the registry is voluntary, all participants and/or their legal representatives signed a written informed consent. ČENDA registry is approved by the Ethical Committee of the Motol University Hospital and registered at the National Bureau for Personal Data Protection.

In ČENDA registry, collected data include basic demographic information, glycemic control status, data on acute or chronic complications and comorbidities and data on the type of treatment modality and continuous glucose monitoring use and their change. CGM usage is further categorized based on the proportion of time the child spent on CGM in the past year: no use,≤19%, 20%-39%, 40%-69%, 70%-89% and ≥90% category (16).

All children with T1D aged <19 years treated with one of the following HCLs - Medtronic MiniMed 780G (780G), Tandem t:slim X2 with Control-IQ algorithm (Control-IQ) or AAPS with Dana Diabecare RS (SOOIL Development, Seoul, Republic of Korea) or Accu-Chek Insight (Roche Diabetes Care, Mannheim, Germany) insulin pump for at least 12 months and monitored by CGM more than 70% of the time were included in the analysis. The study flowchart is shown in the **Supplementary Figure 1**. Before the initiation of HCL therapy, all children were educated about the proper configuration of the system and its appropriate utilization.

The median HbA1c, CGM-derived parameters and Glycemia Risk Index (GRI) from the last available visit were calculated and compared between the HCL groups. CGM-derived parameters included the following parameters: time in range – TIR (3.9-10.0 mmol/L; 70-180 mg/dL); time in hyperglycemia level 1 – TAR1 (10.1-13.9 mmol/L; 181-250 mg/dL); time in hyperglycemia level 2 – TAR2 (>13.9 mmol/L; >250 mg/dL); time in hypoglycemia level 1 – TBR1 (3.0-3.8 mmol/L; 54-69 mg/dL); time in hypoglycemia level 2 – TBR2 (<3.0 mmol/L; <54 mg/dL) (17). The median of the CGM-derived parameters were calculated from the last 14 days’ CGM records before the last outpatient visit. The Glycemia Risk Index was calculated using the standard formula: GRI = (3.0 × TBR <50 mg/dL) + (2.4 × TBR <70 mg/dL) + (1.6 × TAR >250 mg/dL) + (0.8 × TAR >180 mg/dL) (18). The occurrence of severe hypoglycemia (SH) and/or diabetic ketoacidosis (DKA) in 2022 was also collected and compared across the groups. A separate age-category analysis (0–5.99, 6–11.99, and 12-18.99 years) was performed for all of the above-mentioned parameters.

### 2.2 Statistical analysis

Data were summarized as means with standard deviation (SD) or medians with interquartile range where appropriate. The differences between HCL groups were assessed using ANOVA F-test or Kruskall-Wallis ANOVA. Categorical variables were summarized using absolute and relative frequencies and differences between the groups were tested using χ^2^-test. For better insight, cumulative distribution functions for HbA1c, TIR, and GRI were used to examine the relationship between the HCL groups.

To reduce the imbalance of baseline characteristics between the groups, we used the *mnps* function for multiple groups of the TWANG (The Toolkit for Weighting and Analysis of Nonequivalent Groups) library (19) to estimate the propensity score weights based on gender, current age, T1D duration, insulin dose, and BMI. This type of analysis differs from usual propensity score matching, in that it allows for multiple groups to be considered at once and keeps the original sample sizes. Weighted means/medians, 1^st^ and 3^rd^ quartiles were computed to reassess the differences. Comparisons between HCL groups were then carried out using weighted ANOVA regression models and Tukey *post-hoc* analysis for pairwise comparisons.

## 3 Results

### 3.1 Baseline characteristics

Data from 512 CwD (276 males and 236 females) who met the inclusion criteria were analyzed. 780G, Control-IQ and AAPS were used by 217 (42.4%), 211 (41.2%) and 84 (16.4%) children, respectively. The mean age of CwD in the study cohort was 12.8 ± 4.2 years, with the age category 12+ years the most represented (n = 323), followed by children aged 6–11.99 (n = 142) and <6 years (n = 46). The mean diabetes duration was 7.0 ± 3.6 years. We observed a statistically significant difference in age, T1D duration, duration of HCL therapy, daily insulin requirement and BMI-SDS between the users of studied HCL systems. The basic characteristics of the study group are summarized in the **Supplementary Table 1** in detail.

### 3.2 HbA1c

**Figure 1:**
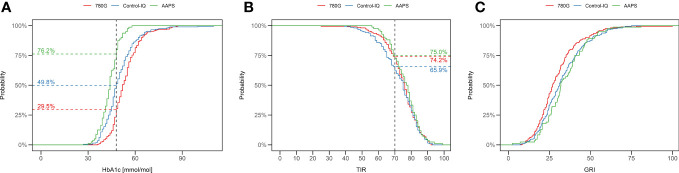
Percentage of children using 780G (red), Control-IQ (blue) and AAPS (green) achieving the ISPAD target of HbA1c 48mmol/mol **(A)**, and TIR (70%) **(B)**. The difference in proportions of CwD reaching a particular GRI value are depicted as C. **(C)** TIR, time in range; GRI, glycemia risk index.

**Figure 2:**
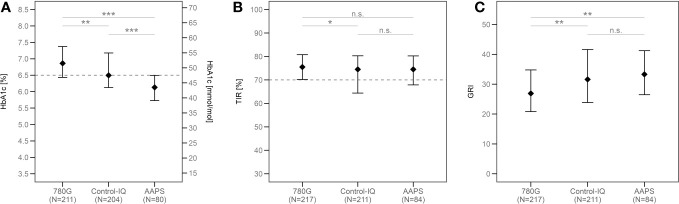
The medians of HbA1c **(A)**, TIR **(B)** and GRI **(C)** in all groups according to the type of HCL system used after the propensity score weighting. ***p<0.001, **p<0.01, *p<0.05, ns, not significant. TIR, time in range; GRI, glycemia risk index; AAPS, AndroidAPS.

The median of HbA1c in the whole study group was 49 mmol/mol (6.6%). The ISPAD target of HbA1c <48 mmol/mol (<6.5%) (20) was reached by 76.2% of AAPS users, 49.8% of Control-IQ users, and 29.5% of 780G users. (**Figure 1A**). The lowest HbA1c value was seen in the AAPS users (44 mmol/mol; 6.2%; p<0.001 vs any other group), followed by the Control-IQ (48 mmol/mol, 6.5%) and 780G group (52 mmol/mol; 6.9%) (p<0.001 between the latter). (**Supplementary Figure 2A**). Similar results of HbA1c were observed after propensity score weighting recalculation (**Figure 2A**) and in all of the evaluated age groups (**Supplementary Figure 3A**; **Supplementary Table 2**).

### 3.3 Times in glycemic ranges

**Figure 3:**
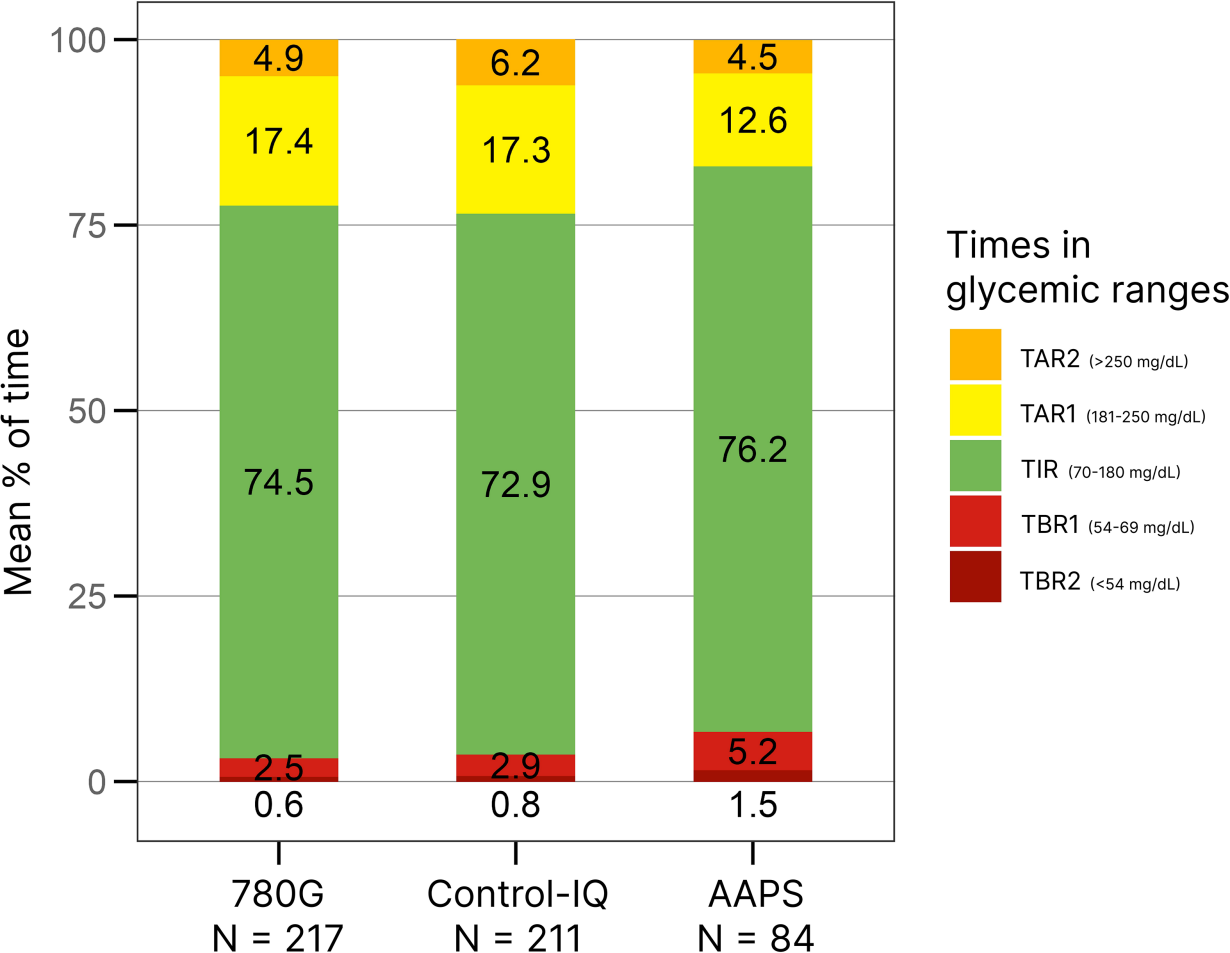
The means of times in glycemic ranges. AAPS, AndroidAPS.

A detailed overview of TIR in the study groups is shown in **Table 1**, the means of TIR are illustrated in **Figure 3**. The recommended target of TIR 70% (20) was achieved by 75% of CwD in the AAPS group, 74.2% of CwD in the 780G group, and 65.9% of CwD in the Control-IQ group. (**Figure 1B**) The highest median of TIR was achieved by AAPS users (78%), followed by 780G (76%), and Control-IQ users (75%). Only the difference between the AAPS and the Control-IQ group was assessed as statistically significant (p=0.035). On the other hand, the AAPS group spent the longest time in hypoglycemia with the mean of TBR1 5.2% (vs 2.9% for Control-IQ and 2.5% for 780G) and TBR2 1.5% (vs 0.8% for Control-IQ and 0.6% for 780G).

**Table 1:** Parameters of glycemic control by the type of HCL used. The results are shown as medians (IQR), and for DKA and SH the results are shown as events per 100-patient years.

	All patients					Recalculation after propensity score weighting				
	780G	Control-IQ	AAPS	Total	p-value	780G	Control-IQ	AAPS	Total	p-value
**HbA1c [mmol/mol]**	52 (48-58)	48 (44-55)	44 (40-48)	49 (44-56)	< 0.001	51 (47-57)	48 (44-55)	44 (39-48)	48 (43-55)	< 0.001
**HbA1c (%)**	6.9 (6.5-7.5)	6.5 (6.2-7.2)	6.2 (5.8-6.5)	6.6 (6.2-7.3)	< 0.001	6.8 (6.5-7.4)	6.5 (6.2-7.2)	6.2 (5.7-6.5)	6.5 (6.1-7.2)	< 0.001
**TIR [%]**	76 (69-81)	75 (65-81)	78 (70-82)	76 (68-81)	0.127	76 (70-81)	75 (65-80)	75 (68-80)	75 (68-81)	0.015
**TAR >180 mg/dL [%]**	17 (13-21)	17 (12-22)	12 (9.0-16)	16 (12-21)	< 0.001	17 (13-20)	17 (12-22)	12 (9.3-17)	16 (1-20)	< 0.001
**TAR >250 mg/dL [%]**	4.0 (1.0-7.0)	4.0 (2.0-8.0)	3.0 (2.0-6.0)	4.0 (2.0-7.0)	0.035	2.5 (0.7-6.1)	3.5 (1.6 -8.0)	3.5 (1.4 - 7.2)	3.5 (1.2 - 6.8)	0.009
**TBR <70 mg/dL [%]**	2.0 (1.0-3.0)	2.0 (1 -4)	4.0 (3.0 -7.0)	3.0 (1.0-4.0)	< 0.001	1.5 (0.6 -3.2)	2.5 (1.0 -3.5)	3.5 (2.2 - 6.7)	2.5 (0.9-4.0)	< 0.001
**TBR <54 mg/dL [%]**	0.0 (0.0-1.0)	1.0 (0.0-1.0)	1.0 (0.0-2.0)	0.0 (0.0-1.0)	< 0.001	0.0 (0 -0.6)	0.5 (0 - 0.8)	0.5 (0 -1.9)	0.0 (0.0 -1.9)	0.009
**GRI**	27 (21-36)	31 (24-41)	32 (26-41)	30 (22-39)	0.001	27 (21 -35)	32 (24 -42)	34 (26 -41)	31 (23 -40)	< 0.001
**GRI hyperglycemia component**	13 (8.5-17)	12.5 (8.2-20)	9.2 (6.5-15)	12 (8.0-18)	< 0.001	12 (8.0-16)	13 (8.4 -20)	9.8 (6.5-16)	12 (7.7-17)	< 0.001
**GRI hypoglycemia component**	1.6 (0.8-3.6)	2.6 (0.9-4.2)	5.2 (2.4-8.1)	2.6 (0.8-4.4)	< 0.001	1.7 (0.5 -3.8)	2.5 (1.1 -4.2)	4.1 (2.2 -8.5)	2.5 (1-4.9)	< 0.001
**DKA**	3.3	2.0	0.0	2.2	NS	NA	NA	NA	NA	NA
**SH**	0.9	2.0	1.2	1.4	NS	NA	NA	NA	NA	NA

NS, non-significant; NA, not available.

AAPS, AndroidAPS; TIR, time in range; GRI, glycemia risk index; DKA, diabetic ketoacidosis; SH, severe hypoglycemia; IQR, interquartile range.

After the recalculation using the propensity score weighting, the similar results were observed in all of the groups, with the TIR of 76% scored by 780G users, 75% by AAPS users and 75% by Control-IQ users. While the TIR of the 780G group did not differ significantly from the AAPS group (p=0.99), there was a statistical difference between the 780G and the Control-IQ group (p=0.02, **Figure 2B**). The medians of TIR in all age groups are shown in detail in **Supplementary Table 2**.

### 3.4 Glycemia Risk Index

The median GRI of all CwD included in the study was 30. The lowest GRI value was achieved by the users of 780G (27), followed by Control-IQ (31) and AAPS (32) (**Table 1**). The difference in GRI between 780G users and the other two assessed HCL systems was significant (p<0.05), whereas no significant difference was found between the Control-IQ and AAPS groups (p=0.72). An overview of GRI results is shown in **Table 1** and **Supplementary Figure 2C**. The cumulative distribution of GRI by HCL systems is shown in **Figure 1C**.

The lowest GRI in the 780G group (p<0.005 vs both other groups) as well as no significant difference between AAPS and Control-IQ users (p=0.53) was consistently observed also in the matched cohort (**Figure 2C**) and all age categories (**Supplementary Figure 3C**; **Supplementary Table 2**).

### 3.5 DKA and severe hypoglycemia

There was no statistically significant difference observed in the occurrence of diabetic ketoacidosis nor severe hypoglycemia events between the groups over the observed study period (**Table 1**).

## 4 Disscusion

This population-based study compared the parameters of glycemic control in CwD treated by one of the HCL systems (MiniMed 780G, Control-IQ, AAPS) for at least one year. The results revealed that all three systems are effective in achieving the international recommended goals of T1D control. Nevertheless, there are clearly discernible differences that illustrate the strengths and weaknesses of the systems assessed.

The results of well-powered pediatric studies testing the HCL systems individually are in line with our data. Arrieta et al. demonstrated a mean of TIR 73.9% in a cohort of 3211 CwD treated with 780G (21). Similarly Breton et al. described TIR 73.5% in a group of 9451 children using Control-IQ (13). These data are comparable with our findings as in our cohort the mean TIR values of 74.5% and 72.9% were recorded for 780G and Control-IQ, respectively.

To date, similarly focused studies are characterized by small number of participants and limited spectrum of outcomes. The 1-month real-life observational study of 31 CwD did not reveal any significant differences in CGM-derived parameters between Control-IQ and 780G (mean TIR 70.5% vs 70.1%) (22). In contrast, Bassi et al. compared these two systems retrospectively in a 1-year follow-up study comprising 74 children and adults with type 1 diabetes and observed a significant superiority of the 780G system in terms of time in range (71% vs 68%, p=0.001), time above range (p=0.001), average glucose levels (p=0.001) and standard deviation of glycemia (p=0.031) (23). The DIY AAPS system has not been subjected to a comparison in similar studies yet.

Our study revealed some differences in the parameters of glucose control between the HCL systems. Generally, AAPS users achieved the lowest HbA1c, however, they also presented with the highest hypoglycemia rates. In contrast, CwD using 780G were characterized by the lowest time spent in hypoglycemia and consequently scored the lowest GRI. The explanation for these differences might lie in the system settings and the algorithms used by the systems. 780G uses a self-adjusting technology and only allows users to set the insulin-to-carbohydrate ratio, target glycemia, and insulin activity. This setting significantly reduces the potential for insulin overdose when hyperglycemia is corrected by the user. This might explain the lowest hypoglycemia rate in the 780G group and consequently, the highest HbA1c value since hypoglycemia is one of the main factors contributing to the HbA1c value (24). Additionally, the 780G scored the lowest GRI underlining the fact that this index is preferentially driven by hypoglycemia rather than hyperglycemia (18).The position of AAPS is on the opposite side of the spectrum as this system enables the user to individualize and adjust any of the settings. Moreover, AAPS is a DIY system that requires the user to initially download and set it up possibly biasing this group with more motivated and tech-savvy CwD and/or their parents/guardians. Given the flexibility of AAPS input settings and the potentially higher motivation of AAPS users to achieve the lowest possible HbA1c, these users may be prone to overcorrect hyperglycemia with a subsequent risk of hypoglycemia. The Control-IQ algorithm represents a kind of middle ground between these systems. Most settings can be adjusted by the user but some functionalities (i.e. target glycemia) can only be changed to a limited extent. Thus, it scores mostly in the middle between 780G and AAPS in the evaluated parameters.

Based on our results, we propose that 780G might be an advantageous option for CwD with recurrent hypoglycemia episodes or CwD with a fear or impaired awareness of hypoglycemia. On the other hand, higher time in hypoglycemia found in the AAPS group suggests that clinicians should preferentially focus on addressing this in CwD treated with this system, possibly adjusting the settings accordingly and emphasizing the risks of hypoglycemia and its prevention.

Our study has several strengths, which encompass a representativeness of the study population (including children younger than 6 years), unique data on AAPS, and a broad spectrum of parameters (including first data on GRI in HCL systems).

There are several limitations of our study. Firstly, there were pre-existing differences between the groups in diabetes duration, age, insulin dose, and BMI-SD. The number of CwD using a specific HCL also differed. To this end, we used propensity score weighting to minimize the bias and enable a meaningful comparison. The results remained similar even after propensity score weighting which might give our findings more credence. However, despite the use of propensity score weighting, we were unable to eliminate the bias stemming from differences in individual device settings, bolus timing, and the correct use of automatic mode by the participants (25). On the other hand, all of the subjects underwent similar standardized education during the introduction of HCL which should minimize this bias. As this is a cross-sectional observational study, we cannot exclude selection bias at the level of individual diabetologists preference of one of the HCL systems. A large number of children included and the propensity score weighting analysis nonetheless mitigates this risk. Additionally, we were not able to include some relevant information that were not collected in the ČENDA database in 2022 such as average glycemia and glycemic variability.

Although all of the tested HCL systems proved effective in maintaining recommended long-term glycemic control, we observed differences that might illustrate strengths and weaknesses of particular systems. Our findings could help individualizing the choice of HCL systems.

## Data availability statement

The raw data supporting the conclusions of this article will be made available by the authors, without undue reservation.

## Ethics statement

The studies involving humans were approved by Ethics committee of the Motol University Hospital, Prague, Czechia. The studies were conducted in accordance with the local legislation and institutional requirements. Written informed consent for participation was not required from the participants or the participants’ legal guardians/next of kin in accordance with the national legislation and institutional requirements.

## Author contributions

AS: Conceptualization, Investigation, Methodology, Project administration, Writing – original draft, Writing – review & editing. LuP: Conceptualization, Investigation, Methodology, Project administration, Writing – original draft, Writing – review & editing. VN: Conceptualization, Investigation, Methodology, Project administration, Writing – original draft, Writing – review & editing. MP: Data curation, Formal Analysis, Methodology, Visualization, Writing – original draft, Writing – review & editing. LeP: Conceptualization, Writing – original draft, Writing – review & editing. PK: Investigation, Writing – original draft, Writing – review & editing. PV: Investigation, Writing – original draft, Writing – review & editing. JaS: Investigation, Writing – review & editing. RP: Investigation, Writing – review & editing. DN: Investigation, Writing – review & editing. JV: Investigation, Writing – review & editing. KK: Investigation, Writing – review & editing. BO: Investigation, Writing – review & editing. SP: Conceptualization, Investigation, Methodology, Project administration, Writing – original draft, Writing – review & editing. OC: Conceptualization, Methodology, Writing – original draft, Writing – review & editing. ZS: Conceptualization, Funding acquisition, Investigation, Methodology, Supervision, Visualization, Writing – original draft, Writing – review & editing. JiS: Investigation, Writing - review & editing.
