# Supplementary material for: Are all HCL systems the same? long term outcomes of three HCL systems in children with type 1 diabetes: real-life registry-based study
Source: Front Endocrinol (Lausanne). 2023 Oct 16;14:1283181. doi: 10.3389/fendo.2023.1283181 (PMC10613700; doi:10.3389/fendo.2023.1283181)
Supplement: Supplementary file 1 [file DataSheet_1.docx]

**SUPPLEMENTARY**

Supplementary Figure 1. The study flowchart. T1D – type 1 diabetes, HCL – hybrid closed loop, CGM – continuous glucose monitoring, AAPS – AndroidAPS

**

**

Supplementary Figure 2. The medians of HbA1c **(A)**, TIR **(B)** and GRI **(C)** in all groups according to the type of HCL system used, and without recalculation using propensity score matching. ***p<0.001, **p<0.01, *p<0.05, ns= not significant. AAPS – AndroidAPS, TIR – time in range, GRI – glycemia risk index


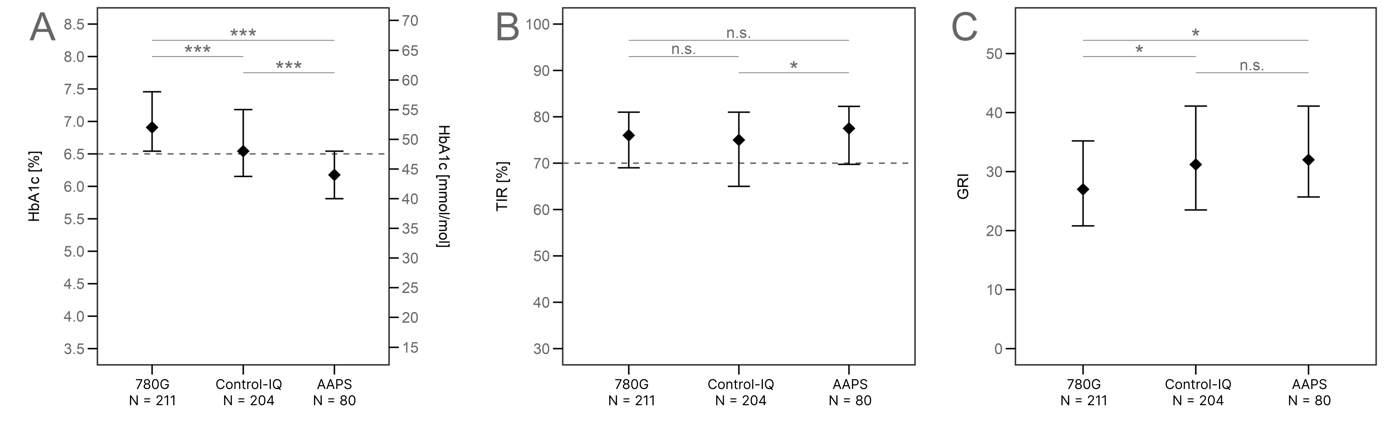


Supplementary Figure 3. The medians of HbA1c **(A)**, TIR **(B)** and GRI **(C)** by age categories.

AAPS – AndroidAPS, TIR – time in range, GRI – glycemia risk index


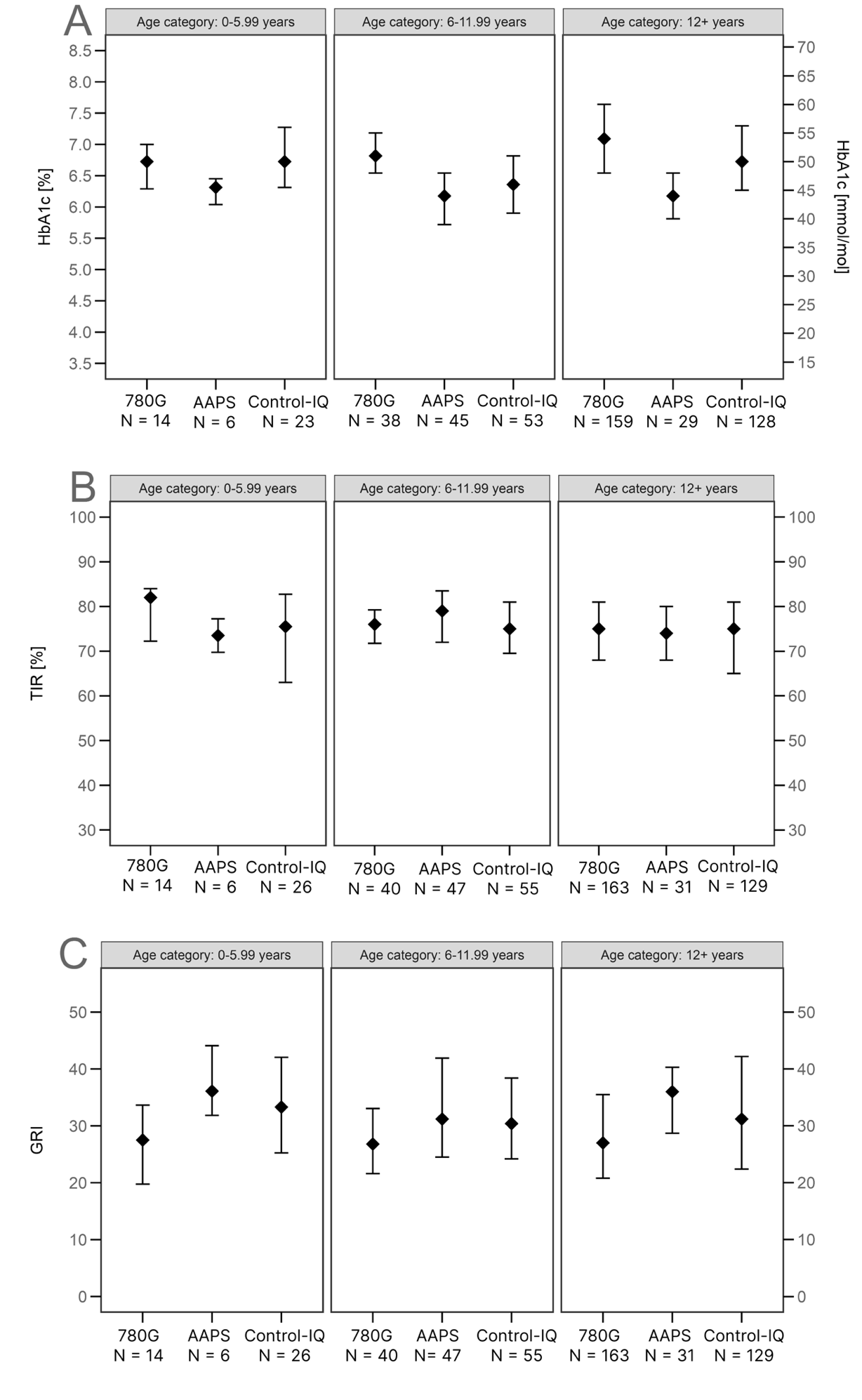


Supplementary Table 1. Basic characteristics of the study group.

AAPS – AndroidAPS

|  | **780G** | **Control-IQ** | **AAPS** | **Total** | **p-value** |
| --- | --- | --- | --- | --- | --- |
| **Gender – male [N (%)]** | 117 (53.9%) | 118 (55.1%) | 41 (48.8%) | 276 (46.1%) | 0.542 |
| **Age [years] mean (SD)** | 13.8 (3.9) | 12.5 (4.4) | 11.0 (3.5) | 12.8 (4.2) | <0.001 |
| **T1D duration**  **[years] mean (SD)** | 8.3 (3.7) | 6.0 (3.5) | 6.5 (2.7) | 7.0 (3.6) | <0.001 |
| **Insulin dose [U/kgBW/day] mean (SD)** | 0.9 (0.2) | 0.8 (0.2) | 0.9 (0.3) | 0.9 (0.2) | 0.046 |
| **BMI-SDS (mean (SD))** | 0.71 (1.3) | 0.70 (1.44) | 0.22 (0.99) | 0.62 (1.33) | 0.009 |
| **HCL therapy duration [years] mean (SD)** | 1.51 (0.34) | 1.71 (0.89) | 2.74 (1.18) | 1.79 (0.88) | <0.001 |

Supplementary Table 2. Parameters of glycemic control by HCL type and age category.

AAPS – AndroidAPS, TIR – time in range, GRI – glycemia risk index

|  | **Age <6 years** | | | | **Age 6-12 years** | | | | **Age > 12 years** | | | |
| --- | --- | --- | --- | --- | --- | --- | --- | --- | --- | --- | --- | --- |
|  | **780G** | **Control-IQ** | **AAPS** | **p-value** | **780G** | **Control-IQ** | **AAPS** | **p-value** | **780** | **Control-IQ** | **AAPS** | **p-value** |
| **Gender – male [N (%)]** | 4 (28.6) | 14 (53.8) | 3 (50.0) | 0.341 | 25 (62.5) | 28 (50.9) | 22 (46.8) | 0.318 | 88 (54.0) | 75 (58.1) | 16 (51.6) | 0.702 |
| **T1D duration**  **[years] mean (SD)** | 2.8 (1.2) | 2.4 (0.8) | 3.3 (0.9) | 0.090 | 5.8 (2.2) | 5.0 (2.3) | 5.8 (1.9) | 0.105 | 9.4 (3.4) | 7.1 (3.6) | 8.2 (3.1) | < 0.001 |
| **Insulin dose [U/kgBW/day] mean (SD)** | 0.74 (0.17) | 0.72 (0.22) | 0.81 (0.24) | 0.606 | 0.90 (0.21) | 0.76 (0.2) | 0.80 (0.25) | 0.015 | 0.89 (0.23) | 0.88 (0.24) | 0.95 (0.25) | 0.283 |
| **BMI-SDS (mean (SD))** | 0.47 (0.96) | 0.62 (1.37) | 0.23 (0.41) | 0.746 | 0.42 (1.19) | 0.33 (1.06) | -0.10 (0.89) | 0.049 | 0.81 (1.35) | 0.88 (1.57) | 0.68 (1.06) | 0.771 |
| **HbA1c median [mmol/mol] (IQR)** | 50 (45 -53) | 50 (45 - 56) | 46 (43 -47) | 0.287 | 51 (48 -55) | 46 (41 -51) | 44 (39 - 48 | < 0.001 | 54 (48 - 60) | 50.(45 - 56) | 44(40 - 48) | < 0.001 |
| **TIR median [%] (IQR)** | 82(72 - 84) | 76 (63 - 83) | 74 (70 - 77) | 0.360 | 76 (72 - 79) | 75 (70 – 81) | 79 (72 - 84) | 0.221 | 75 (68 - 81) | 75(65 - 81) | 74 (68 - 80) | 0.545 |
| **TAR> 180 mg/dL median [%] (IQR)** | 13 (9.2 - 19) | 16 (11 - 21) | 14 (12 - 15) | 0.696 | 17 (13 - 19) | 15 (11 - 20) | 11.0 (8.5-15) | < 0.001 | 18 (14 – 22) | 18 (13 - 24) | 12 (10 - 18) | 0.005 |
| **TAR> 250 mg/dL median [%] (IQR)** | 1.5 (1.0 – 5.0) | 4.0 (2.0 - 11) | 5.5 (2.8 - 9.8) | 0.063 | 3.5 (1.0-7.0) | 4.0 (2.5-7.0) | 3.0 (1.5-4.5) | 0.085 | 4.0 (1.0 – 7.0) | 4.0 (2.0 – 9.0) | 5.0 (2.0 – 8.0) | 0.212 |
| **TBR< 70 mg/dL median [%] (IQR)** | 3.5 (3.0 - 5.8) | 3.0 (1.2 - 4.8) | 4.0 (3.2 - 4.8) | 0.185 | 2.0 (1.8 - 3.2) | 3.0 (2.0-4.0) | 5.0 (3.0-7.0) | < 0.001 | 2.0 (1.0 – 3.0) | 2.0 (1.0 – 4.0) | 4.0 (2.0 – 7.0) | < 0.001 |
| **TBR< 54 mg/dL median [%] (IQR)** | 1.0 (0.0-1.0) | 0.0 (0.0 - 1.8) | 1.0 (1.0-1.0) | 0.614 | 0.0 (0.0-1.0) | 1.0 (0.0-1.0) | 2.0 (0.0-2.5) | < 0.001 | 0.0 (0.0-1.0) | 0.0 (0.0-1.0) | 1.0 (0.0-2.0) | 0.0559 |
| **GRI median(IQR)** | 28 (20 - 34) | 33 (25 - 42) | 36 (32 - 44) | 0.217 | 27 (22 - 33) | 30 (24 - 38) | 31(25 - 42) | 0.133 | 27 (21 - 36) | 31 (22 - 42) | 36 (29 - 40) | 0.021 |
| **GRI hyperglycemia component (IQR)** | 7.2 (6.0 - 14) | 12 (6.5 - 23) | 13(8.6 - 20) | 0.183 | 13(8.5 - 15) | 12(8.5 - 16) | 8.0 (5.5-11) | 0.002 | 13(8.5 - 19) | 13 (8.5 - 22) | 12 (8.2-16) | 0.341 |
| **GRI hypoglycemia component (IQR)** | 3.3 (2.4 - 5.6) | 2.9 (1.0 - 5.2) | 4.2 (3.6 - 6.3) | 0.235 | 2.0 (1.4 - 3.7) | 3.4 (1.6-5.0) | 5.8 (3.0 - 8.5 | < 0.001 | 1.6 (0.8-3.4) | 2.4 (0.8-4.0) | 4.2 (2.1 - 8.2) | < 0.001 |
| **DKA [events per 100-patient years]** | 0.0 | 0.0 | 0.0 | NS | 5.0 | 0.0 | 0.0 | NS | 3.2 | 3.3 | 0 | NS |
| **SH [events per 100-patient years]** | 7.1 | 0.0 | 0.0 | NS | 0.0 | 0.0 | 0.0 | NS | 0.6 | 3.3 | 3.2 | NS |
